# Preeclampsia in Saudi Arabia: A Systematic Review and Meta-Analysis of Prevalence, Risk Factors, Awareness, and Outcomes

**DOI:** 10.7759/cureus.98969

**Published:** 2025-12-11

**Authors:** Rwdyn R Nujoom, Raghad B Babader, Albatul A Aloufi, Mohammed Malibary, Nouf H Alshareef, Fatema M Shoaib, Afrah Almteri, Leen S Al Maqwashi, Maria D Alrafi

**Affiliations:** 1 Obstetrics and Gynecology, King Abdulaziz University Hospital, Jeddah, SAU; 2 Internal Medicine, Ministry of Health, Jeddah, SAU; 3 Obstetrics and Gynecology, Sulaiman Alrajhi University, Qassim, SAU; 4 Medicine, Almaarefa University, Riyadh, SAU

**Keywords:** adverse outcomes, antenatal care, awareness, fetal health, maternal health, preeclampsia, prevalence, public health initiatives, risk factors, saudi arabia

## Abstract

This systematic review aimed to evaluate the prevalence, awareness, risk factors, and outcomes of preeclampsia among women in Saudi Arabia.In accordance with the Preferred Reporting Items for Systematic Reviews and Meta-Analyses guidelines, we searched PubMed, Web of Science, and Cochrane databases, focusing on studies published between 1999 and 2024. We included observational studies conducted in Saudi Arabia reporting the risk factors, prevalence, and outcomes of preeclampsia.Overall, 21 studies were included in the analysis, of which 15 analyzed prevalence. The prevalence of preeclampsia in Saudi Arabia was 4.80%, with higher rates reported in cross-sectional studies (9.52%). Only 27% of women demonstrated preeclampsia awareness, and the key identified risk factors included serum lead, immunoglobulin A nephropathy, and calcium, magnesium, and zinc deficiencies. Adverse maternal outcomes included hemolysis, elevated liver enzymes, and low platelet syndrome and eclamptic seizures, whereas fetuses faced significant risks, including stillbirth, prematurity, and intrauterine growth restriction (IUGR).Preeclampsia poses a significant health burden in Saudi Arabia, which is compounded by limited public awareness and critical risk factors. These findings underscore the urgent need for improved preeclampsia screening and education in expectant mothers. Enhanced antenatal care and public health initiatives targeting preeclampsia awareness may improve maternal and fetal outcomes and reduce the long-term health burdens associated with this condition.

## Introduction and background

Hypertension is one of the most common chronic public health concerns worldwide, including in Saudi Arabia, and it significantly contributes to morbidity and mortality. It is associated with various implications, including cardiovascular disease, stroke, and renal failure. Preeclampsia is a particularly severe hypertensive disorder of pregnancy due to its potential for devastating maternal and fetal consequences. It is defined as new-onset hypertension after 20 weeks of gestation (blood pressure ≥ 140/90 mmHg on two occasions at least four hours apart) in a previously normotensive woman, accompanied by either proteinuria or, in the absence of proteinuria, new-onset maternal organ dysfunction such as thrombocytopenia, impaired liver function, renal insufficiency, pulmonary edema, or new-onset neurological symptoms [1,2]. Current evidence emphasizes that proteinuria is not required for diagnosis, highlighting the importance of systemic features in clinical evaluation [1,3].

Although the exact pathophysiology remains under investigation, preeclampsia is widely understood as a two-stage disorder. The first stage involves abnormal placentation with inadequate trophoblastic invasion and poor remodeling of the spiral arteries, leading to placental hypoxia [4]. In the second stage, the dysfunctional placenta releases anti-angiogenic and inflammatory mediators that trigger widespread maternal endothelial dysfunction and vasoconstriction [4].

Globally, preeclampsia affects approximately 2%-8% of pregnancies, making it a significant contributor to maternal and perinatal morbidity and mortality [5]. In Saudi Arabia, the incidence is 2.4% of pregnancies [6]. While maternal mortality from preeclampsia is rare in developed countries, it remains a leading cause of intensive care unit (ICU) admissions during pregnancy and contributes to long-term health risks for both the mother and child [7]. Women with a history of hypertensive disorders in pregnancy, pre-existing conditions, such as diabetes or autoimmune diseases, advanced maternal age, obesity, or multifetal pregnancies are at higher risk of developing preeclampsia [6]. The 2019 National Institute for Health and Care Excellence guidelines recommend low-dose aspirin (75-150 mg daily) for women with significant risk factors, as its prophylactic use reduces the incidence of preeclampsia when initiated between 12 and 16 weeks of gestation and continued until 36 weeks of gestation [1,7].

Awareness of preeclampsia among pregnant women is crucial for early recognition and timely management. Studies in Saudi Arabia have shown variable levels of awareness regarding symptoms, risk factors, and consequences, with many women unaware of the importance of blood pressure monitoring and warning signs such as persistent headaches, visual disturbances, or sudden swelling of the hands and face [8]. Healthcare providers play a critical role in patient education, particularly for women at elevated risk. Maternal complications include increased long-term risk of cardiovascular disease, stroke, and chronic hypertension [9]. Fetal complications commonly include preterm birth, fetal distress, and intrauterine growth restriction (IUGR), and emerging evidence suggests potential long-term metabolic and cardiovascular risks for exposed offspring [10].

As our understanding of preeclampsia grows, further research is needed to identify predictive biomarkers, novel therapeutic strategies, and long-term health outcomes for affected mothers and children. The aim of this systematic review is to synthesize existing evidence on the prevalence, risk factors, awareness, and maternal and fetal outcomes of preeclampsia in Saudi Arabia. 

This article was previously presented at the Saudi Society of Women’s Health, Top House Conferences on November 29-30, 2024.

## Review

Materials and methods

Protocol and Registration

This systematic review was prospectively registered in PROSPERO (ID: CRD42024525920) and adhered to the 2020 Preferred Reporting Items for Systematic Reviews and Meta-Analyses (PRISMA) guidelines [11]. The review was performed between January 2024 and January 2025. The PROSPERO registration was checked prior to conducting this review.

Eligibility Criteria

We included observational studies (cross-sectional, cohort, and case-control) conducted in Saudi Arabia that reported at least one of the following outcomes: prevalence of preeclampsia, awareness of preeclampsia, maternal outcomes (e.g., eclampsia, hemolysis, elevated liver enzymes, and low platelets (HELLP) syndrome, ICU admission, and maternal mortality), fetal outcomes (e.g., preterm birth, low birth weight, and perinatal mortality), and associated risk factors or prevention strategies.

Studies were eligible if participants were pregnant women diagnosed with preeclampsia according to the diagnostic criteria applied in each study (e.g., American College of Obstetricians and Gynecologists (ACOG) and International Society for the Study of Hypertension in Pregnancy (ISSHP)).

We excluded review articles, conference abstracts without full text, editorials, case reports/series, non-human studies, studies conducted outside Saudi Arabia, studies not reporting relevant quantitative data, and studies published in languages other than English.

Information Sources and Search Strategy

A comprehensive electronic search was conducted in PubMed/MEDLINE, Web of Science, Cochrane Library, and Google Scholar from database inception to March 2025. The search strategy used a combination of MeSH terms and free-text keywords related to preeclampsia and Saudi Arabia. The core search string applied was as follows: (“preeclampsia” OR “pre-eclampsia” OR “hypertensive disorders of pregnancy”) AND (“Saudi Arabia” OR “Kingdom of Saudi Arabia”). Boolean operators (AND/OR) were used to refine the search for each database. The full database-specific search strategies are provided in the Appendices to ensure replicability.

Study Selection

All retrieved records were uploaded into Rayyan, where duplicate studies were removed automatically. Two reviewers independently screened the titles and abstracts in Rayyan using predefined inclusion and exclusion criteria, followed by full-text review [12]. Disagreements at any stage were resolved through discussion.

Screening Criteria

All pregnant women meeting the criteria for preeclampsia, defined as blood pressure exceeding 140/90 mmHg (with two readings taken four hours apart), urine albumin levels exceeding 1, those with hemolysis, elevated liver enzymes, and low platelets (HELLP) syndrome, and those experiencing accelerated hypertension in known cases of hypertension (systolic blood pressure > 170 mmHg or diastolic blood pressure > 120 mmHg, or new-onset proteinuria), were screened [1,2].

Data Collection Process

Two reviewers independently extracted data into a standardized form. Extracted variables included study characteristics (first author, year, study period, design, setting, and journal); sample size (total and number with preeclampsia); participant demographics (age, gestational age, and parity); preeclampsia prevalence and awareness; risk factors, prevention measures, and management strategies; and maternal and fetal outcomes.

Missing or unclear data were recorded as reported; no AI-assisted tools were used to retrieve missing information.

​​​​*Risk of Bias Assessment*

The methodological quality of all included studies was assessed using the Joanna Briggs Institute (JBI) Critical Appraisal Tools, appropriate for cross-sectional, cohort, and case-control designs. Each study was evaluated across key domains, including selection, measurement, comparability, and reporting, and then categorized as low, moderate, or high risk of bias according to JBI guidance (Table 1).

**Table 1:** JBI scale scores showing the methodological quality of the included studies JBI: Joanna Briggs Institute

Author ID	Study design	JBI score	Quality category	Risk of bias
Al-Jameil et al. [13]	Case-control	8	Good	Low
Al-Jameil [14]	Cohort	7	Good	Low
Al Jameil [15]	Cohort	8	Good	Low
Gari et al. [3]	Cross-sectional	7	Good	Low
Alshayeb et al. [16]	Cross-sectional	8	Good	Low
Sobande et al.[17]	Retrospective cohort	7	Good	Low
Aljuaid et al.[18]	Case-control	8	Good	Low
Subki et al.[6]	Retrospective cohort	5	Fair	Moderate
Mousa et al. [2]	Retrospective	8	Good	Low
Al-Ghamdi et al. [19]	Retrospective	7	Good	Low
Al-Mulhim et al. [20]	Retrospective cohort	6	Fair	Moderate
Waness et al. [21]	Prospective case series	3	Poor	High
Al-Jameil et al. [22]	Prospective case-controlled	7	Good	Low
Bahkali et al.[8]	Cross-sectional	8	Good	Low
Alfaqih and Al-Saiali [23]	Descriptive cross-sectional	6	Fair	Moderate
Osman et al.[24]	Cross-sectional	8	Good	Low
Radwan et al.[25]	Cross-sectional	6	Fair	Moderate
Sadaruddin et al.[26]	Descriptive study	8	Good	Low
El-Tahan et al.[27]	Randomized trial	8	Good	Low
Ali et al. [28]	Case-control	7	Good	Low
Gull et al. [29]	Case-control	7	Good	Low

Data Synthesis and Statistical Analysis

Descriptive analyses of the included studies were performed to summarize their characteristics and main findings. A comprehensive systematic review and meta-analysis approach was used. All forest plots and quantitative meta-analyses were conducted using RevMan 5.4 statistical software (The Cochrane Collaboration, London, UK).

Prevalence and awareness proportions were synthesized using raw, untransformed data. A random-effects model using the DerSimonian-Laird estimator was applied due to anticipated heterogeneity among included studies.

A pooled analysis was conducted to estimate the prevalence of preeclampsia toxemia (PET). Heterogeneity was assessed using Cochran’s Q-test, the I² statistic, and Tau², all automatically generated within RevMan. Subgroup analyses were performed to explore potential sources of heterogeneity based on study design and primary population characteristics. Forest plots were used to visually present the prevalence and awareness of PET among Saudi women. A separate pooled analysis was carried out to assess PET awareness, with subgroup analyses focusing on symptoms, risk factors, and complications. All pooled estimates were reported with 95% confidence intervals (95% CIs). Both qualitative and quantitative methods were applied to synthesize the existing evidence on PET in Saudi Arabia.

Sensitivity analyses were not performed, as all studies meeting the eligibility criteria were retained in the final meta-analysis. This has been acknowledged as a methodological limitation.

Results

Search Results

**Figure 1:**
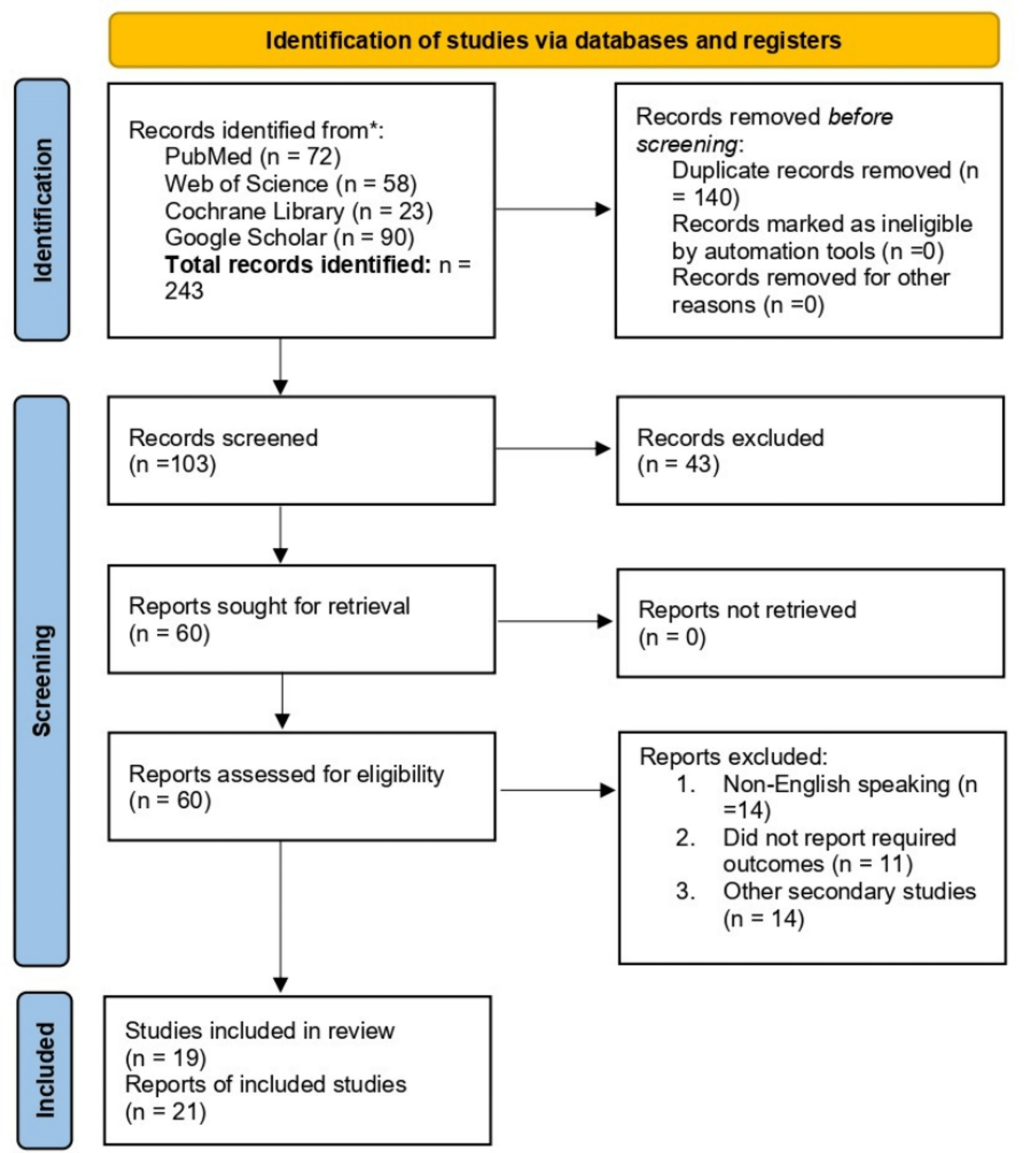
PRISMA flow diagram summarizing the search strategy PRISMA: Preferred Reporting Items for Systematic Reviews and Meta-Analyses

The online search yielded a total of 243 records. After removing 140 duplicates, 103 records remained for title and abstract screening. Of these, 43 records were excluded. Sixty full-text articles were retrieved and assessed for eligibility. Forty-one articles were excluded for the following reasons: non-English language (n = 14), lack of required outcomes (n = 11), and secondary studies such as systematic reviews and meta-analyses (n = 14). Ultimately, 19 studies (21 reports) met the inclusion criteria and were included in the final systematic review. Figure 1 presents a PRISMA diagram summarizing the search strategy.

Characteristics of the Included Studies

The total sample size of our study was 42,296 women of reproductive age, ranging from 15 to 45 years. Among expectant women, gestation ranged from the first trimester (6-12 weeks) to the third trimester (≥28 weeks). The characteristics of the included studies are summarized in Table 2 and Table 3.

**Table 2:** Characteristics of the included studies

Author ID	Study design	Setting	Sample size (% with preeclampsia toxemia)	Age (mean/range)	Gestational age (weeks)	Parity
Al-Jameil et al. (2017) [13]	Case-control study	Riyadh, Saudi Arabia	120 (33.3%)	31.55 ± 6.14	33.72 ± 3.70	Not reported
Al-Jameil (2014) [14]	Cohort study	Riyadh, Saudi Arabia	120 (33.3%)	31.55 ± 6.14	33.72 ± 3.70	Not reported
Al Jameil (2014) [15]	Cohort study	Riyadh, Saudi Arabia	120 (33.3%)	31.55 ± 6.14	33.72 ± 3.70	Not reported
Gari et al. (2022) [3]	Cross-sectional study	Makkah, Saudi Arabia	378 (11.1%)	>35 years	21.98 ± 13.31	28.5% were nulliparous
Alshayeb et al. (2024) [16]	Cross-sectional study	Al-Asha, Saudi Arabia	390	27.1 ± 12.8	1st trimester, 16; 2nd trimester, 20; 3rd trimester, 14	46% of the currently pregnant women were in their first pregnancy, and 44.6% of the participants had no previous pregnancies, while 25.6% had 1-2 previous pregnancies
Sobande et al. (2007) [17]	Retrospective cohort study	Southwest region of Saudi Arabia	315 (94.3%)	29.0 + 6.0	34.28 + 5.1	72.2% of the eclamptic patients were nulliparous
Aljuaid et al. (2020) [18]	Case-control	Jeddah, Saudi Arabia	233	30.6 ± 5.7	Not reported	Does not specify
Subki et al. (2018) [6]	Retrospective cohort	Jeddah, Saudi Arabia	9,493	31.3 ± 6.7	25-41	Does not specify
Mousa et al. (2022) [2]	Retrospective	Jeddah, Saudi Arabia	83	32 ± 6.28	33.32 weeks and 36 weeks	Average parity was 3
Al-Ghamdi et al. (1999) [19]	Retrospective	Northwestern, Saudi Arabia	208	27 ± 6	Not reported	60% of the participants were primigravid (first pregnancy), whereas the rest had one or more pregnancies
Al-Mulhim et al. (2003) [20]	Retrospective cohort study	Dammam, Saudi Arabia	27,787	Not reported	<37 weeks, 30.2%; >37 weeks, 69.8%	Does not specify
Waness et al. (2010) [21]	Prospective case series	Jeddah, Saudi Arabia	12	28.6 ± 3.5	≥28 weeks	Sixty-three patients (30.3%) were primigravida, whereas 96 (46%) were grand multiparous (>5)
Al-Jameil et al. (2014) [22]	Prospective case-controlled study	Riyadh, Saudi Arabia	120	± 6.14	≥24 weeks	42% were nulliparous (first pregnancy), 45.3% had 1-4 previous pregnancies, and 12.7% had 5 pregnancies
Bahkali et al. (2024) [8]	Cross-sectional	Jeddah, Saudi Arabia	541	34.5 ± 8.7	Not reported	31.6% of the participants had never been pregnant
Alfaqih and Al-Saiali (2017) [23]	Descriptive cross-sectional	Taif, Saudi Arabia	200	33.6 ± 4.7	1st trimester, 6; 2nd trimester, 74; 3rd trimester, 118	62% of women had 5-12 previous pregnancies, and 47% had 5-11 previous deliveries
Osman et al. (2023) [24]	Cross-sectional	Al Baha, Saudi Arabia	485	≥40	34-37	40.5% were nulliparous (first pregnancy), whereas 59.5% had previous pregnancies
Radwan et al. (2023) [25]	Cross-sectional	Jeddah, Saudi Arabia	1,169	25.94 ± 7.94	Not reported	Does not specify
Sadaruddin et al. (2020) [26]	Descriptive study	Buraidah Al-Qassim, Saudi Arabia	143	Not reported	Not reported	Does not specify
El-Tahan et al. (2018) [27]	Randomized trial	Al Khubar, Saudi Arabia	38	Not reported	≥34 weeks	60% of the women were multiparous, whereas the rest were nulliparous
Ali et al. (2018) [28]	Case-control	Riyadh, Saudi Arabia	179	29.3	6-12 weeks	Does not specify
Gull et al. (2020) [29]	Case-control	Jeddah, Saudi Arabia,	162	28.34 ± 5.53	35.37 ± 3.18	Does not specify

**Table 3:** Characteristics of the included studies BUN: blood urea nitrogen, Cr: creatinine, HDL: high-density lipoprotein, HELLP: hemolysis, elevated liver enzymes, and low platelets, HR: high risk, IgA: immunoglobulin A, IUGR: intrauterine growth restriction, IV: intravenously, LDL-C: low-density lipoprotein cholesterol, NICU: neonatal intensive care unit, PE: pulmonary embolism, RCTs: randomized controlled trials, SNPs: single nucleotide polymorphisms, VLDL-C: very low-density lipoprotein cholesterol

Author ID	Risk factors	Prevention measures	Treatment	Maternal outcomes	Fetal outcomes
Al-Jameil et al. (2017) [13]	Mean serum levels of calcium, magnesium, and zinc were lower in the HR group and even lower in the preeclampsia group than in the control group	Normal levels of trace elements in the serum may prevent preeclampsia	Not reported	Serum levels of trace elements (calcium, magnesium, and zinc) and their correlation with preeclampsia, blood pressure, and other biochemical markers	Not reported
Al-Jameil (2014) [14]	Maternal serum lead levels	Not reported	Not reported	Maternal serum lead levels were significantly higher in the preeclampsia group, correlating with elevated systolic and diastolic blood pressure; the study identifies lead as a risk factor for preeclampsia	Not reported
Al Jameil (2014) [15]	Higher urinary protein and BUN:Cr ratio in the HR group could be identified as risk factors for preeclampsia and subsequent renal disorder	The use of 24-hour urinary protein as an early predictor will help to identify pregnant females at high risk of preeclampsia and prompt the initiation of education and prophylactic interventions (i.e., primary prevention, e.g., close prenatal care)	Hyperproteinuria with hyperuricemia and high BUN:Cr ratio correlate with severe preeclampsia, and an increase in BUN:Cr ratio in the HR group is an indicator for an effective prophylactic treatment to prevent the onset of renal damage in pregnant women	High blood pressure, acute renal failure, and complications related to renal dysfunction	Intrauterine growth restriction, prematurity, and fetal death
Gari et al. (2022) [3]	Advanced maternal age, obesity, and/or vascular disorders	Not reported	Delivery of the infant and placenta	Maternal complications noted in the study include acute kidney injury, liver damage, and premature delivery	Not reported
Alshayeb et al. (2024) [16]	Family history of preeclampsia, having prior preeclampsia, diabetes, history of clotting problems, obesity, unhealthy lifestyle, multiple births, and family history of pregnancy poisoning	Awareness of preeclampsia and paying attention to warning signs such as headaches, vision changes, and swelling, and reporting them to their healthcare providers immediately	Not reported	51.8% reported renal dysfunction as a complication, and 51.3% knew about maternal death as a potential complication of preeclampsia	Fetal death
Sobande et al. (2007) [17]	The only independent risk factor for eclampsia was the presence of prodromal symptoms	Not reported	-	-	-
Aljuaid et al. (2020) [18]	Not reported	Not reported	Not reported	No significant associations were found between the SNPs and the risk of developing preeclampsia in the Saudi population	Not reported
Subki et al. (2018) [6]	Advanced maternal age and personal and family histories of PE were more prevalent in women with PE, gestational hypertension, or diabetes; however, the association was significant only in women with diabetes in pregnancy	Screening for this disorder is recommended early in pregnancy	Not reported	Maternal complications were reported in 9.4% of the women, and the prevalence of maternal mortality was 1.3%	Fetal weight (mean: 2,683 g) and Apgar scores at 1 minute (mean: 7.4) and at 5 minutes (mean: 8.7)
Mousa et al. (2022) [2]	Not reported	Early screening and taking a patient’s medical history, measuring blood pressure, and providing consistent and effective management according to international and national guidelines	Not reported	Eclampsia, hypertension, HELLP syndrome, hydralazine-induced lupus, and maternal complications	Intrauterine fetal demise, neonatal death, prematurity, IUGR, respiratory distress syndrome, neonatal jaundice, and low birth weight
Al-Ghamdi et al. (1999) [19]	Not reported	Not reported	Oral antihypertensive medications, diazepam IV during labor, injectable antihypertensive medications, combination therapy, and magnesium sulfate infusion	Four patients developed eclampsia	Perinatal mortality
Al-Mulhim et al. (2003) [20]	Nulliparous, extreme age	Better antenatal care, early recognition of PE, and delivery at the appropriate time	Not reported	Placental abruption was the most common maternal complication, followed by oliguria, coagulopathy, and renal failure	Stillbirths and early neonatal deaths
Waness et al. (2010) [21]	IgA nephritis is a risk factor for preeclampsia	Not reported	Pregnancies in patients with IgA nephritis require close observation	Hypertension, preeclampsia, and one patient developed HELLP syndrome	No fetal complications
Al-Jameil et al. (2014) [22]	Abnormal lipid profile, increased triglycerides and LDL-C levels, and decreased levels of good cholesterol (HDL)	Not reported	Not reported	Increase in triglycerides, LDL-C, VLDL-C, and proteinuria, indicating higher susceptibility to cardiovascular disorders and preeclampsia complications	Not reported
Bahkali et al. (2024) [8]	Advanced age group, nulliparity	All women should receive a good education about the signs and symptoms of preeclampsia	Not reported	Severe kidney or liver failure, pulmonary edema, cerebral hemorrhage, and disseminated intravascular coagulation	Preterm birth, IUGR, placental abruption, and fetal death in utero
Alfaqih and Al-Saiali (2017) [23]	Age, age at first pregnancy, and weight gain	Proper screening, medical education, and health education sessions should be conducted for all females, especially pregnant women, about the hazards associated with maternal age, high parity, high gravida, habitual abortions, hypertension, diabetes mellitus, etc.	Not reported	Anemia, gestational diabetes, and pregnancy-induced hypertension	18.2% of neonates were admitted to the NICU, 3% had low birth weight, and 3% experienced neonatal death
Osman et al. (2023) [24]	Obesity, diabetes mellitus, chronic hypertension, chronic kidney disease, kidney disorders, heart disorders, and preterm delivery	By bridging the existing knowledge gaps and empowering women with the necessary information, healthcare providers and policymakers can work toward improving maternal and fetal health outcomes	Not reported	Severe hypertension, kidney failure, pulmonary edema, and maternal death	Preterm birth, IUGR, and stillbirth
Radwan et al. (2023) [25]	Regarding preeclampsia risk factors, 27.3% agreed that a family history of preeclampsia is a risk factor, while 60.3% agreed that having prior preeclampsia is a risk factor; the frequency of agreement on other preeclampsia risk factors was as follows: obesity (58%), diabetes (64.2%), unhealthy lifestyle (70%), and multiple births (19.2%)	One effective approach is to use social media and community events to disseminate information and raise awareness about the risks and symptoms of preeclampsia; we can educate more women about this potentially life-threatening condition by contacting diverse population segments; it is also essential to work with community leaders, influential individuals, and women’s associations to promote awareness and empower women to take charge of their health by seeking appropriate medical care, including prenatal services	Not reported	Maternal deaths	Fetal growth restriction, small-for-gestational-age infants, and prematurity
Sadaruddin et al. (2020) [26]	Extreme reproductive age	Efforts should be made to reduce the risk factors responsible for the high incidence of preeclampsia and eclampsia at the grass-roots level; awareness and resources should be made available at all levels to reduce the maternal and fetal complications	Not reported	Severe hypertension, proteinuria, and placental abruption	Stillbirths, prematurity, and growth retardation
El-Tahan et al. (2018) [27]	Not reported	Not reported	Compared with dexmedetomidine (0.4 g/kg/h), the preoperative administration of remifentanil (0.1 g/kg/min) produced a significantly higher effect on maternal hemodynamic responses to cesarean delivery in patients with severe preeclampsia; however, maternal hypotension and neonatal respiratory depression were more common with the use of remifentanil	Severe hypertension, preeclampsia, and placental abruption leading to an increased rate of emergency cesarean deliveries	Low birth weight, preterm birth, and neonatal respiratory distress syndrome
Ali et al. (2018) [28]	Not reported	Not reported	A daily dose of 4,000 IU of vitamin D reduces the risk of preeclampsia in vitamin D-deficient pregnant women; this is an economical, safe, and easily correctable intervention to combat harmful conditions such as preeclampsia and IUGR; additional RCTs are stipulated based on the fact that related RCTs are heterogeneous and inconclusive; screening of vitamin D3 deficiency and its treatment is recommended for favorable obstetric outcomes	The incidence of preeclampsia was 1.2% in group 2 (4,000 IU) compared with 8.6% in group 1 (400 IU)	The study reports fewer cases of IUGR in the group receiving higher doses of vitamin D, with IUGR present in 9.6% of group 2 compared with 22.2% in group 1
Gull et al. (2020) [29]	Not reported	Not reported	For I/D polymorphism, no significant differences were detected in the genotype and allele frequencies or any of the inheritance models between patients with preeclampsia and controls (Arab women)	Hypertension, proteinuria, and renal and hepatic involvement	Fetal growth restriction and prematurity

Methodological Quality of the Included Studies

The Joanna Briggs Institute (JBI) Critical Appraisal Tools were used to determine methodological quality. Only one study had poor quality, namely, Waness et al. [21], which had a small sample size. Other studies had either fair or good methodological quality.

Preeclampsia Prevalence

**Figure 2:**
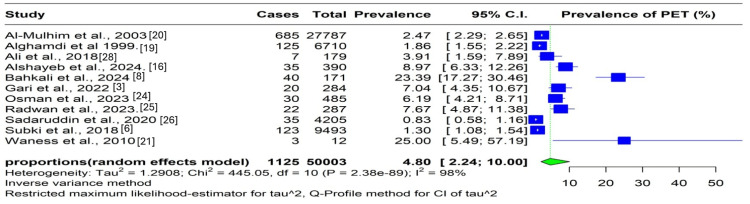
Forest plot showing preeclampsia prevalence among women of reproductive age in Saudi Arabia CI: confidence interval, PET: preeclampsia toxemia

Ten studies reported preeclampsia prevalence among women of reproductive age in different parts of Saudi Arabia. A pooled analysis of reported outcomes showed that the aggregate preeclampsia prevalence in Saudi Arabia was 4.80% (95% CI: 2.24%, 10.00%) (Figure 2). However, heterogeneity was very high (I2 = 98%), necessitating the use of a random-effects model to pool the summary effect sizes. We performed a subgroup analysis to explore the sources of heterogeneity. The first subgroup analysis was based on study design. We sought to determine whether differences would be observed if participants were enrolled over time (longitudinal studies) or at a certain point in time (cross-sectional studies). Our subgroup analysis revealed that the aggregate prevalences were 9.52% (95% CI: 4.64, 18.53) in cross-sectional studies and 2.59% (95% CI: 0.75%, 8.56%) in longitudinal studies. However, overall heterogeneity across the groups remained very high (91% and 95%, respectively).

Then, we sought to determine if the primary study population affected prevalence since certain studies used women of reproductive age as the primary population, whereas others used pregnant women with gestational hypertensive disease.

Chronic (pre-existing) hypertension is not classified as pregnancy-induced hypertension (PIH). To ensure adherence to accepted definitions of hypertensive disorders in pregnancy, women with pre-existing hypertension were considered as having chronic hypertension rather than PIH. Because chronic hypertension is a known risk factor for preeclampsia, its inclusion in some study populations may influence the observed prevalence. To minimize misclassification bias, we used the total number of pregnant women during the study period as the denominator in the two studies that reported overall deliveries, regardless of whether women had chronic hypertension or gestational hypertension. This approach ensured that preeclampsia prevalence was calculated using an appropriate and consistent population base across studies [19,25]. Other studies exclusively included women with gestational hypertension; therefore, we performed a subgroup analysis to explore whether this would explain the heterogeneity. The subgroup analysis revealed that the preeclampsia incidences were 4.18% (95% CI: 1.97, 8.66) in an inclusive population and 25% (95% CI: 1.97, 8.660) in the population with gestational hypertension. However, heterogeneity across studies remained high (I2 = 98% in the former) (Appendices).

Preeclampsia Risk Factors

A quantitative synthesis of the correlation between various risk factors and preeclampsia incidence was not possible owing to inconsistent reporting of results. Therefore, we conducted a systematic review of these studies, which investigated different preeclampsia risk factors. Al-Jameil et al. investigated the relationship between serum trace elements and the risk of developing preeclampsia [13], reporting that reduced levels of trace elements, e.g., zinc, calcium, and magnesium, were associated with preeclampsia pathogenesis. Furthermore, other studies showed that increased serum lead levels and blood urea nitrogen (BUN):creatinine ratios were risk factors for preeclampsia development [14,15]. In studies investigating genetic predisposition, none of the mutations were found to be predisposing factors for preeclampsia [18,28]. Finally, Waness et al. reported that patients with immunoglobulin A (IgA) nephropathy had a high predisposition for preeclampsia, requiring close observation during pregnancy [21].

Preeclampsia Outcomes

Only a few studies reported maternal and fetal outcomes of preeclampsia; therefore, a meta-analysis of these outcomes was not feasible. Reported preeclampsia outcomes in mothers included eclamptic seizures, ICU admission, placental abruption, coagulopathy, and HELLP syndrome [6,18,20]. Main adverse fetal outcomes included stillbirth, neonatal death, prematurity, and IUGR [25].

Level of Preeclampsia Awareness

**Figure 3:**
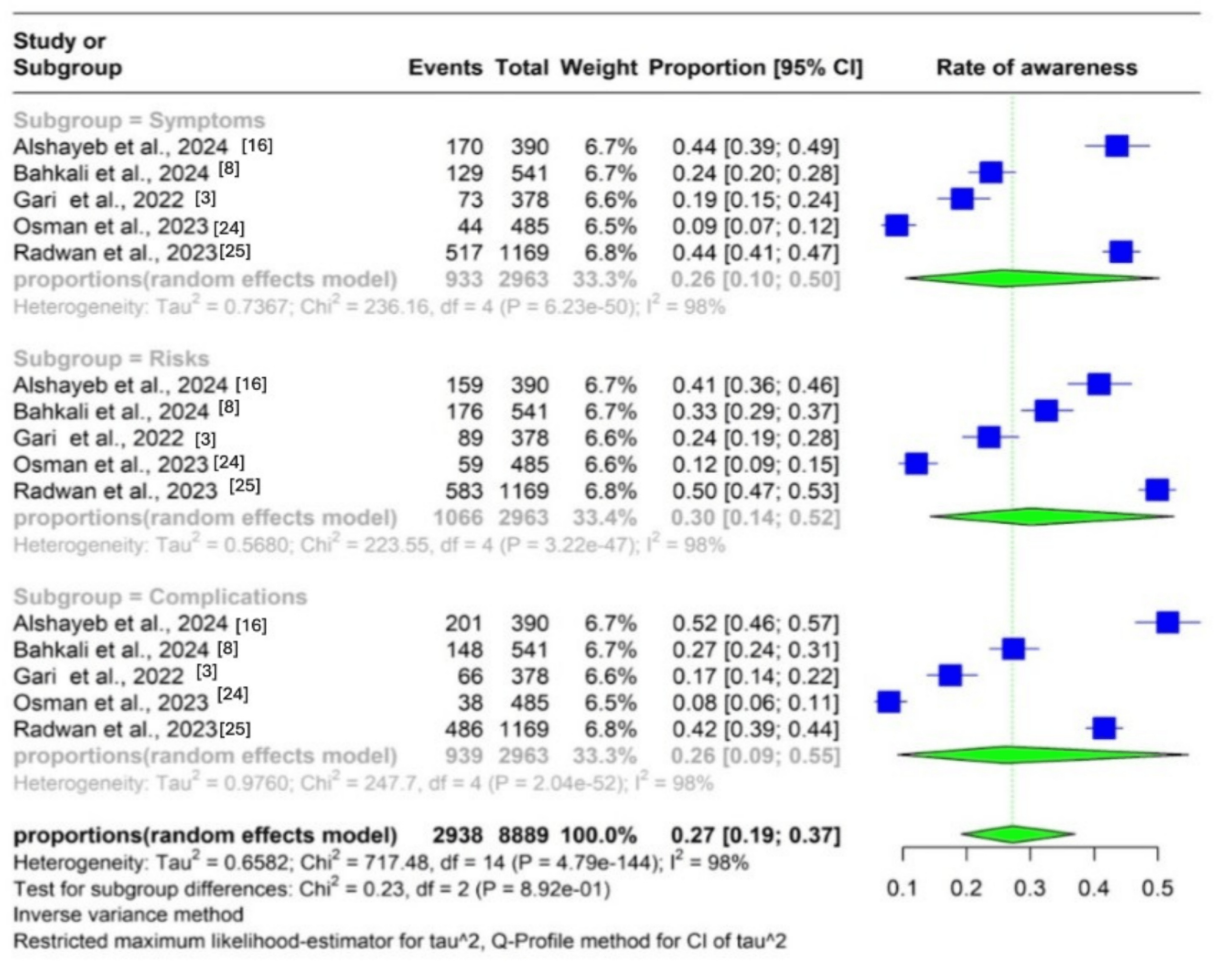
Forest plot showing the level of preeclampsia awareness among women of reproductive age in Saudi Arabia CI: confidence interval, PET: preeclampsia toxemia

Five studies reported the level of preeclampsia awareness. A pooled analysis of the reported outcomes showed that 27% (95% CI: 19%, 44%) of women in Saudi Arabia had some preeclampsia awareness. The subgroup analysis showed that 26% of them (95% CI: 10, 50) were aware of preeclampsia symptoms, 30% (95% CI: 14%, 52%) of preeclampsia risk factors, and 26% (95% CI: 9%, 55%) of preeclampsia complications. The analysis showed high heterogeneity across all subgroups (I2 = 98%; Figure 3).

Discussion

Preeclampsia Prevalence

The overall frequency of preeclampsia in women of reproductive age in Saudi Arabia was 4.80%. Cross-sectional studies had higher prevalence rates than longitudinal studies. Preeclampsia incidence was considerably higher in studies that included 25% of the population with gestational hypertension than in those that did not.

Preeclampsia Risk Factors

A comprehensive analysis revealed several possible preeclampsia risk factors, including decreased levels of trace elements, e.g., calcium, magnesium, and zinc, and elevated BUN:creatinine ratios and serum lead levels. No significant correlation was observed between preeclampsia and genetic predispositions, such as certain mutations. IgA nephropathy was another important risk factor necessitating careful monitoring throughout pregnancy.

Preeclampsia Outcomes in Mothers and Fetuses

Common maternal outcomes included coagulopathy, placental abruption, HELLP syndrome, eclamptic seizures, and ICU admission, whereas main fetal outcomes included prematurity, IUGR, stillbirth, and neonatal death.

Preeclampsia Awareness

Overall, 27% of women in Saudi Arabia were aware of preeclampsia, 26% knew about preeclampsia symptoms, 30% knew about risk factors, and 26% knew about preeclampsia complications. We observed a lack of consistent information among communities, as evidenced by the wide variations in awareness levels among studies.

References to Relevant Literature

The findings of this systematic review and meta-analysis are consistent with those of several studies. For example, Al-Jameil et al. found an association between decreased serum levels of trace elements, e.g., zinc, magnesium, and calcium, and preeclampsia pathogenesis, in line with the observed risk factors [13]. Similarly, Gari et al. revealed gaps in community knowledge regarding preeclampsia symptoms and complications, reinforcing our finding that only 27% of women demonstrated preeclampsia awareness [3].

Subki et al. [6] and Osman et al. [24] identified significant preeclampsia risk factors, including advanced maternal age, obesity, and chronic diseases, such as diabetes, which are consistent with our findings. These two studies further emphasized the heightened susceptibility of women with pre-existing conditions. The study by Waness et al. supports our findings regarding the role of specific comorbidities, e.g., IgA nephropathy, in predisposing women to preeclampsia [21].

Mousa et al. identified maternal complications, such as HELLP syndrome, placental abruption, and ICU admissions, as significant preeclampsia outcomes. This study further highlighted adverse fetal outcomes, e.g., prematurity and IUGR [2]. Rahnemaei et al. suggested preventive interventions, such as aspirin and micronutrient supplementation, to enhance antenatal care and promote targeted prevention strategies [30].

Implications on Clinical Practice

Pregnancy monitoring and planning in specialized centers involve screening pregnant women for risk factors of hypertensive disorders, identifying high-risk women early in pregnancy, and initiating preventive therapy. A retrospective study conducted in 2022 examined other preventive measures for preeclampsia and sought to investigate how well blood parameters predict the severity of gestational hypertension and preeclampsia using blood samples from participants before 20 weeks’ gestation. There was a significant difference in the aminotransferase:platelet ratio index (APRI) value between the control and severe preeclampsia groups. The platelet:lymphocyte ratio, neutrophil:lymphocyte ratio, delta neutrophil index, and platelet distribution width were not clinically significant. The previous study was the first to examine the relationship between the APRI and pregnancy-related hypertension, with significant implications for clinical practice and the literature [31].

This systematic review details the effects of preeclampsia on the mother and fetus. Early screening and preventive actions are necessary since preeclampsia compromises maternal health during pregnancy and presents the risk of long-term multisystemic problems. Low-molecular-weight heparin, enoxaparin, pentaerythritol tetranitrate, yoga, and micronutrients, e.g., L-arginine, folic acid, and vitamin D, alone or in combination with calcium, were found to prevent preeclampsia in a systematic review published in 2020. However, findings on aspirin use remain debatable [30].

A study conducted in 2021 concluded that implementing broader definitions of preeclampsia may lead to a higher incidence of disease diagnosis. The previous study aimed to investigate the effects of using three different preeclampsia diagnostic criteria on the rates of disease diagnosis, disease severity, and adverse outcomes, and determine correlations between each component of the various diagnostic criteria and unfavorable pregnancy outcomes. However, whether this would result in better outcomes remains unclear since women who meet the new criteria may have a milder disease phenotype [32].

Comparison to the Current Gold Standard of Care

Recent research on hypertensive disorders during pregnancy suggests significant advancements that may enhance the current gold standard of care. The existing approach involves routine monitoring of blood pressure and proteinuria after 20 weeks of gestation and is heavily dependent on patient history for risk assessment. However, emerging insights emphasize the importance of early identification of high-risk women and the use of predictive markers such as the APRI. Incorporating these markers may contribute to more proactive and individualized screening protocols.

Regarding preventive interventions, standard care often recommends aspirin for high-risk women starting in the first trimester, along with lifestyle modifications and calcium supplementation. New findings indicate additional effective measures, including low-molecular-weight heparin and micronutrients, suggesting that a broader range of options can be integrated into preventive strategies to better address diverse risk profiles [33].

The diagnostic criteria for preeclampsia currently rely on elevated blood pressure and proteinuria after 20 weeks of gestation and may benefit from a reevaluation. Studies have proposed adopting broader definitions that could increase the diagnosis rates. However, this raises concerns regarding the potential overdiagnosis of milder cases, highlighting the need for a more nuanced approach to ensure that treatment remains appropriate and effective [34].

Additionally, current follow-up care for women with a history of preeclampsia focuses on cardiovascular monitoring. This new research underscores the necessity of structured and tailored long-term follow-up care, emphasizing regular screening for cardiovascular risks and educating women about their heightened vulnerability post-pregnancy [35].

While the current gold standard of care for managing hypertensive disorders in pregnancy is well-established, integrating recent research findings may lead to substantial improvements. Advancements in risk assessment, preventive strategies, diagnostic criteria, and long-term care may significantly improve maternal and fetal health outcomes, emphasizing the importance of continual updates to clinical guidelines [36].

Limitations

This review has several strengths, including the inclusion of numerous studies with diverse populations surveyed through a random stratified sampling technique from various locations and settings. Moreover, our study spanned different generations, encompassed a wide range of behaviors and clinical conditions, and ensured that the diagnosis of preeclampsia was made by trained professionals. Nevertheless, some limitations must be noted.

We included studies with varying levels of bias. Most of the studies were of good quality, representing a low risk of bias. However, four studies were classified as fair quality, indicating a moderate risk of bias, whereas one study was classified as poor quality, indicating a high risk of bias. Moreover, some respondents to the awareness and control questionnaires in certain studies may have been affected by recall bias. The presence of biased studies may skew the overall results and conclusions drawn from this review. Future meta-analyses in this field may benefit from more stringent selection criteria to ensure the inclusion of studies with a lower risk of bias, thereby enhancing the validity and trustworthiness of the results.

Another limitation is the variability in study design and data reporting. The included studies exhibited persistently high heterogeneity, even within the subgroup analyses, which may have affected result comparability. Consequently, this high heterogeneity raises concerns regarding the robustness and generalizability of the findings, causing challenges in drawing definitive conclusions.

This review also did not include a search of grey literature sources such as conference abstracts, dissertations, or government reports. Therefore, some relevant unpublished studies may have been missed, which could introduce publication bias into the review.

Sensitivity analyses were not conducted, as all eligible studies were retained in the final meta-analysis. This limits our ability to determine how specific studies or methodological variations may have influenced the pooled estimates.

Finally, there was the inconsistent reporting of results across the included studies, which hindered the quantitative synthesis of the correlation between different risk factors and preeclampsia incidence. Therefore, we could only conduct a systematic review, rather than a meta-analysis. Moreover, the variability in the reported preeclampsia outcomes poses challenges, with only a few studies detailing maternal and fetal outcomes. This limitation restricts our ability to draw comprehensive conclusions regarding risk factors and outcomes associated with preeclampsia. Furthermore, we aimed to include data on preeclampsia prevention and treatment in this meta-analysis, which was not possible owing to limitations in the extracted data. Future meta-analyses should prioritize standardizing the reporting of risk factors, outcomes, prevention, and treatment related to preeclampsia, ensuring that all relevant studies provide consistent measures and data to facilitate comprehensive quantitative synthesis.

## Conclusions

This systematic review and meta-analysis provided critical insights into preeclampsia prevalence among women of reproductive age in Saudi Arabia, revealing a notable prevalence rate of 4.8%, with cross-sectional studies showing a higher rate than longitudinal studies. Alarmingly, only 27% of the women demonstrated preeclampsia awareness, underscoring a substantial gap in public health education requiring urgent attention. Key risk factors, including elevated serum lead, IgA nephropathy, and mineral deficiencies, underscore the need for early screening and prevention. Severe maternal complications (HELLP syndrome, ICU admission, and coagulopathy) and fetal complications (stillbirth, prematurity, and IUGR) emphasize the importance of improved antenatal care.

Future research should focus on long-term outcomes, biochemical and genetic risk factors, and standardized reporting to enhance prevention and improve maternal-fetal health in Saudi Arabia.

## Figures and Tables

**Figure 4:**
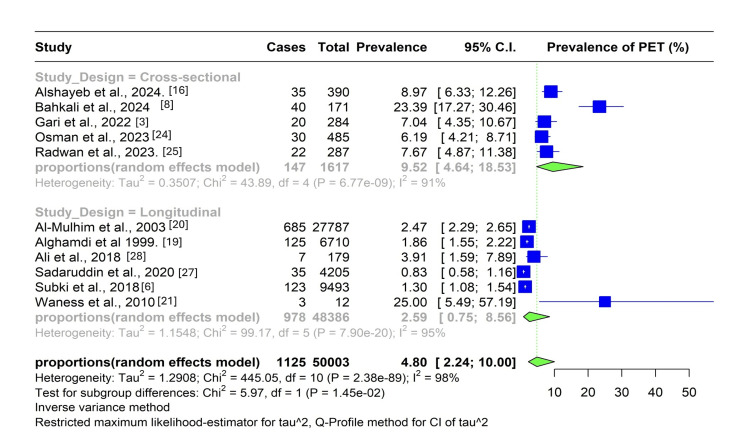
Forest plot showing a subgroup analysis according to the study design of the primary studies CI: confidence interval, PET: preeclampsia toxemia

**Figure 5:**
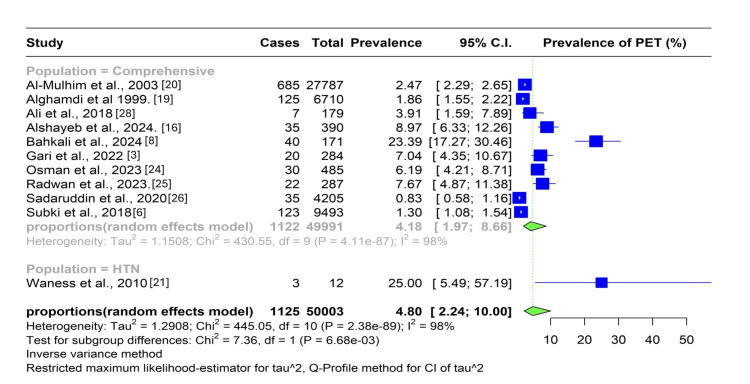
Forest plot showing a subgroup analysis of the prevalence of PET according to the primary study population CI: confidence interval, PET: preeclampsia toxemia

## Appendices

Table 4 provides the full database-specific search strategies used in the present study. 

**Table 4:** Full database-specific search strategies

Database	Search strings
PubMed/MEDLINE	(preeclampsia OR pre-eclampsia OR hypertensive disorders of pregnancy) AND (Saudi Arabia OR Kingdom of Saudi Arabia OR KSA) AND (prevalence OR risk factors OR awareness OR knowledge OR outcomes OR maternal outcomes OR fetal outcomes)
Web of Science	TS=(“preeclampsia” OR “pre-eclampsia” OR “hypertensive disorders of pregnancy”) AND TS=(“Saudi Arabia” OR “KSA” OR “Kingdom of Saudi Arabia”) AND TS=(“prevalence” OR “risk factors” OR “awareness” OR “knowledge” OR “outcomes” OR “maternal outcomes” OR “fetal outcomes”)
Cochrane Library	(“preeclampsia” OR “pre-eclampsia” OR “hypertensive disorders of pregnancy”) AND (“Saudi Arabia” OR “KSA”) AND (“prevalence” OR “risk factors” OR “awareness” OR “outcomes”)
Google Scholar	(“preeclampsia” OR “pre-eclampsia” OR “hypertensive disorders of pregnancy” AND “Saudi Arabia” OR “KSA” AND “prevalence” OR “risk factors” OR “awareness” OR “maternal outcomes” OR “fetal outcomes”)

Figure 4 and Figure 5 present forest plots showing a subgroup analysis according to the study design of the primary studies and of the prevalence of PET according to the primary study population, respectively.
